# Stakeholder Perspectives on Retention Strategies for Rehabilitation Professionals: A Qualitative Study

**DOI:** 10.1177/10497323241286387

**Published:** 2024-12-12

**Authors:** Susanne Mak, Matthew Hunt, Saleem Razack, Kelly Root, Aliki Thomas

**Affiliations:** 1 School of Physical & Occupational Therapy, 12367 McGill University, Montréal, QC, Canada; 2 Institute of Health Sciences Education, McGill University, Montréal, QC, Canada; 3 Centre de recherche interdisciplinaire en réadaptation du Montréal métropolitain, Institut universitaire sur la réadaptation en déficience physique de Montréal (Lindsay Pavillon), Montréal, QC, Canada; 4 Department of Pediatrics, University of British Columbia, BC Children’s Hospital, Vancouver, BC, Canada; 5 Centre for Health Education Scholarship, University of British Columbia, Vancouver, BC, Canada; 6 School of Communication Sciences and Disorders, 12361 Dalhousie University, Halifax, NS, Canada

**Keywords:** occupational therapy, physical therapy, speech-language pathology, retention, rehabilitation, qualitative research, health workforce

## Abstract

There is a scarcity of health human resources worldwide. In occupational therapy (OT), physical therapy (PT), and speech-language pathology (S-LP), attrition and retention issues amplify this situation and contribute to the precarity of health systems. Therefore, we aimed to investigate retention strategies for rehabilitation professionals in Quebec. We present an analysis from individual interviews with rehabilitation professionals and focus groups with stakeholders. We used purposeful sampling (maximum variation approach) to recruit participants from Quebec, Canada. We conducted interviews with 51 OTs, PTs, and S-LPs (2019–2020) and four focus groups with managers, professional education programs, professional associations, and regulatory bodies (2022). Cultural-historical activity theory provided the theoretical scaffolding for these interpretive description studies. Inductive and deductive approaches and constant comparative techniques were used for data analysis. Five sets of retention strategies were developed: (1) ensuring that work aligns with values; (2) improving alignment of work parameters with needs and interests of rehabilitation professionals; (3) modifying physical, social, cultural, and structural aspects of a workplace; (4) addressing how the profession is governed; and (5) offering informal and formal benefits. Multi-systemic retention strategies with intersectoral partnerships were deemed essential to effectively change rehabilitation professionals’ work and work environments and to increase public awareness of the added value of rehabilitation professionals. Our findings emphasize a critical need to design targeted, multi-systemic retention strategies to influence the work experiences of rehabilitation professionals and to ensure the availability of OTs, PTs, and S-LPs for present and future rehabilitation needs.

## Introduction

Attrition is an important concern across health care professions (Heinen et al., 2013; Itzick & Kagan, 2017), including those in rehabilitation (occupational therapy (OT), physical therapy (PT), and speech-language pathology (S-LP)) (World Health Organization [WHO], 2023). Defined as a permanent departure from one’s profession, or from the workforce (McLaughlin et al., 2010), attrition can substantially compromise a health care workforce and, in turn, undermine a health care system’s capacity to deliver patient care (Kroezen et al., 2015).

Attrition from rehabilitation professions has been reported in countries such as Canada, Australia, and the United Kingdom, but the proportions vary across countries and professions. For example, in 2014, 10%–15% of Canadian OTs, PTs, and S-LPs were reported to have left their profession within 2 years of graduation (Ministère de la Santé et des Services Sociaux, 2014). In a 4-year (2000–2004) study in Australia, 65% of surveyed PT graduates from Curtin University planned to leave their profession in the next 10 years (Mulcahy et al., 2010). In a 2023 report from the Royal College of Occupational Therapists (United Kingdom), 25% of surveyed OTs intended to stop working as an OT practitioner in the next 5 years, and less than half anticipated to stop working as an OT after 10 years of practice (Royal College of Occupational Therapists, 2023).

To date, very few studies have investigated the factors that contribute to attrition. In the early 1990s, two survey studies of American OTs identified four main factors that influenced clinicians’ decision to leave the profession: heavy caseloads of patients with multiple comorbidities; experiences of stress and burnout; desire for increased salary and promotional opportunities; and discrepancies between clinician expectations of practice versus actual OT practice (Bailey, 1990; Freda, 1992). Similar findings were identified in a 2010 survey of Australian S-LPs (McLaughlin et al., 2010). Respondents described family commitments and a desire for increased pay and career advancement opportunities as the main reasons for leaving their profession (McLaughlin et al., 2010). In a more recent study, Huddler and Kimmel (2020) interviewed 15 former PTs in the United States and identified several elements that contributed to professional attrition. First, participants expressed frustration with high caseloads and poor quality of care along with poor working conditions. Participants also described how working with managers who were insensitive to their needs and had narrow perspectives on patient care led them to consider leaving their profession, as did negative relationships with colleagues (e.g., lack of respect for them and their work). Finally, a lack of funding for continuing professional development was reported to be especially problematic for PTs who are required to pursue professional development for licensure (Huddler & Kimmel, 2020).

**Figure 1.**
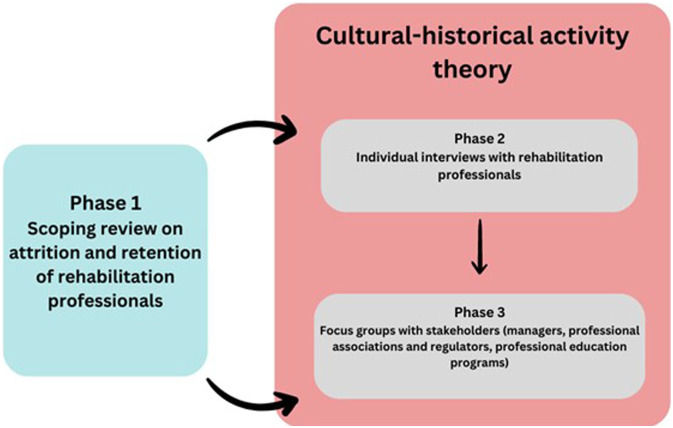
Study phases of the project.

Notwithstanding these four studies, the available evidence on attrition and retention of rehabilitation professionals is limited in scope and specificity. Therefore, we conducted a large three-phase program of research to investigate why OTs, PTs, and S-LPs choose to stay in or leave their profession, and the factors that influence their choices. The research questions were: What factors (personal, professional, and contextual) influence the decision-making of OTs, PTs, and S-LPs in Quebec, to stay in or leave their profession? What are possible strategies to support their retention? Figure 1 presents the study phases and how they relate together. The current paper reports on the findings from phases 2 and 3.

In phase 1, we conducted a scoping review (Mak, Hunt, et al., 2023) to synthesize the literature on attrition and retention in OTs, PTs, and S-LPs. The question guiding the review was: “What is known about attrition and retention in rehabilitation professions?” We retained 59 of the 2588 papers (published from 2010 to 2021) screened for full-text review and extraction. We organized our findings into three themes: (1) descriptions of attrition and retention; (2) experiences of being a professional; and (3) experiences in institutions where rehabilitation professionals work. We identified push, pull, and stay factors at the level of the individual, work, and environment which shaped attrition and retention. *Push* factors propel a rehabilitation professional out of their profession (e.g., negative work experiences) (Mak, Hunt, et al., 2023). Factors that *pull* a rehabilitation professional away from the profession include personal circumstances or a desire to pursue other interests. Finally, *stay* factors, such as positive work relationships and environments, contribute to rehabilitation professionals remaining in their profession (Mak, Hunt, et al., 2023). Our review of this literature suggested that there is a need to closely examine *push* factors (Mak, Hunt, et al., 2023). Recent increased work demands due to the COVID-19 pandemic and the general decline in health workers’ well-being have intensified the urgency to address these factors (Teoh et al., 2023). Given the current workforce shortages in rehabilitation professions globally (WHO, 2023), there is a pressing need to investigate how attrition contributes to these shortages and how this situation influences health care systems’ capacity to deliver high quality care to patients, their families, and communities.

In phase 2, we conducted a qualitative study to further explore the role of push factors in professional attrition and strategies that target such factors. We conducted individual interviews with 51 rehabilitation professionals in Quebec, Canada. We identified six factors that influenced our participants’ work experiences and their decisions to stay in or leave their profession. These findings are reported elsewhere (Mak, Thomas, et al., 2023).

In the third and final phase of our research program, we aimed to explore retention strategies for rehabilitation professionals in Quebec through focus groups with stakeholders. We sought stakeholder (managers, professional education programs, professional associations, and regulatory bodies) perspectives to (1) develop an understanding of their views and experiences related to attrition and retention and (2) generate potential retention strategies for Quebec OTs, PTs, and S-LPs. Retention strategies are interventions aimed at reducing an individual’s intent to leave their profession and can target any level of an organization or system (e.g., individual, group, and organizational) (Teoh et al., 2023). Development of targeted retention strategies is essential to addressing the specific push factors that contribute to attrition among rehabilitation professionals (De Vries et al., 2023).

In this paper, we present an analysis related to retention strategies drawing on individual interviews with rehabilitation professionals (phase 2) and focus groups with stakeholders (phase 3).

## Methods

### Quebec Context

Our empirical investigation focused on OTs, PTs, and S-LPs in Quebec, the province with the second largest population in Canada (Statistics Canada, 2023). Human resource shortages and resource deficiencies have been longstanding issues in Quebec (Denis et al., 2021). These challenges have been so widespread that they have compromised the Quebec health care system’s capacity to deliver adequate care to the population (Gabet et al., 2023). A striking example is the more than 8000 deaths in long-term care facilities during the COVID-19 pandemic, partly due to human resource shortages (Gabet et al., 2023). Unfortunately, Quebec is not alone in experiencing health workforce challenges; its situation is representative of many high-income regions (e.g., United States and United Kingdom) and other Canadian provinces (Casey, 2023).

The Institute of Fiscal Studies and Democracy (2017) reported that successive Quebec governments had under-invested in health care, falling below fundamental health care cost drivers, such as population growth and inflation. While the report highlighted reduced health care spending from 2010 to 2014, there has been a pattern of underspending in health care since the 1990s in Quebec (Institute of Fiscal Studies and Democracy, 2017).

For these reasons, Quebec’s health care context provided an opportune testing ground to conduct our study.

### Design

We used interpretive description (ID) methodology to explore participant perceptions of possible retention strategies. ID is a qualitative research methodology with a naturalistic approach to inquiry. Researchers who use ID aim to examine a phenomenon by identifying common patterns in human experiences while attending to individual differences (Thorne, 2016). Nursing researchers developed ID as an alternate approach to traditional qualitative approaches and as a means for responding to practice-oriented questions in applied health professions (Thorne, 2016). Thus, when using ID, researchers may draw from qualitative traditions (e.g., phenomenology and grounded theory) based on the methodological needs of their research question and typically use clinical and empirical knowledge as a starting point to their empirical inquiry (Thompson Burdine et al., 2021; Thorne, 2016). For these reasons, ID aligned well with our empirical inquiry: we were able to draw from existing literature and our experiences as clinicians and scholars interested in professional practice. Our goal of developing findings with application potential for practice environments was also supported by the theoretical underpinnings of ID, and our inquiry of rehabilitation professionals aligned with ID’s emphasis on disciplinary relevance (Thorne, 2016).

### Theoretical Underpinning

Our study was informed by cultural-historical activity theory (CHAT). CHAT offers a framework to describe a system where several components interact, leading to the implementation of activities which allows the system to reach its intended objective (object) (Engeström, 2001). These components include (1) the main person(s) involved in the activity (subject); (2) the values, norms, guidelines, and expectations that the person must follow to engage in the activity (rules); (3) others whom the person engages with or who contribute to the activity (community); (4) how the activity is shared among the subject and the community (division of labor); and (5) the physical and symbolic objects that influence the activity and are used to complete the activity (tools) (Engeström, 2001). Note that we used the third iteration of CHAT for our inquiries where two activity systems share an object and the unit of analysis is an activity system (Larsen et al., 2019).

We selected CHAT because it offers a broad perspective on systems rather than focusing only on the actors of each system and considers the connections between systems (Larsen et al., 2019; Qureshi, 2021). Specifically, we framed the health care and educational systems as activity systems that shape practitioner experience and, ultimately, a practitioner’s decision to stay in or leave their profession. We defined the delivery of health care services as the object. We also identified other systems, such as the private health sector and professional regulation, as activity systems and considered how they might interact with health care and educational systems to achieve the shared object of health care delivery. These possible interconnections between activity systems underpinned our decision to seek the perspectives of stakeholders from various sectors within and beyond health care (educational system, professional regulation).

### Participants and Recruitment

We used purposive sampling with a maximum variation approach (Suri, 2011) to recruit members from four groups: (1) rehabilitation professionals; (2) managers (public and private health sectors); (3) professional education programs; and (4) professional associations and regulatory bodies. For the rehabilitation professionals and managers, we sent emails to the professional education programs which shared details of our study with their clinical sites and we used social media platforms to announce our study. To recruit participants who had left the profession, we used our personal and professional contacts and a snowball sampling strategy. For the other stakeholder groups, we sent emails to the directors of the professional education programs and to the executive directors of the associations and regulatory bodies.

#### Interviews With Rehabilitation Professionals

We recruited individuals who were previously or currently licensed as OTs, PTs, and S-LPs. More details on the study eligibility criteria are presented in Table 1 and in Mak, Thomas, et al. (2023).

**Table 1.** Inclusion Criteria for Phase 2 Interviews With Rehabilitation Professionals.

Attrition group	• Past member of one of the professional regulatory bodies (*Order of Occupational Therapists of Quebec*, *Professional Order of Physiotherapy of Quebec*, or *Order of Speech-Language Pathologists and Audiologists of Quebec*);
	• Worked for a minimum of 1 year as an OT, PT, or S-LP in Canada;
	• Completed their professional degree in OT, PT, or S-LP in Canada.
Retention group	• Current member of one of the professional regulatory bodies (*Order of Occupational Therapists of Quebec*, *Professional Order of Physiotherapy of Quebec*, or *Order of Speech-Language Pathologists and Audiologists of Quebec*);
	• Worked for a minimum of 1 year as an OT, PT, or S-LP in Canada;
	• Completed their professional degree in OT, PT, or S-LP in Canada.

Participants were divided into two groups: attrition and retention. The attrition group was composed of past members of their professional regulatory body, as licensure is required for practice in Canada (Association of Canadian Occupational Therapy Regulatory Organizations, 2023; Canadian Alliance of Physiotherapy Regulators, 2019; Speech-Language & Audiology Canada, 2023). The retention group included those who were still members of their regulatory body; this group was not further divided into subgroups. We aimed for approximately 15 participants in each group based on existing literature on sample sizes for this type of qualitative inquiry (Hennink & Kaiser, 2022).

One participant was still a member of their regulatory body but because they intended to not renew their membership, they were subsequently placed in the attrition group.

#### Focus Groups With Managers, Professional Associations and Regulatory Bodies, and Professional Education Programs

We recruited (1) managers of rehabilitation professionals from public and private health care sectors, and school settings; (2) representatives from Quebec OT, PT, and S-LP professional education programs; and (3) representatives from professional associations and regulatory bodies. To participate in the study, participants had to meet the inclusion criteria provided in Table 2.

**Table 2.** Inclusion Criteria for Phase 3 Stakeholder Focus Groups.

Managers	• Be a manager, for a minimum of 1 year, of a program/service that currently employs rehabilitation professionals (OT, PT, and/or S-LP)
Professional education programs	• Be the program or associate program director, for a minimum of 1 year, of one of the following university programs:
	• OT: McGill University, University of Montréal, Laval University, University of Québec at Trois-Rivières, University of Sherbrooke
	• PT: McGill University, University of Montréal, Laval University, University of Sherbrooke
	• S-LP: McGill University, University of Montréal, Laval University, University of Québec at Trois-Rivières
Professional associations and regulatory bodies	• Be an employee and a registered member of at least one of the following organizations:
	• Order of Occupational Therapists of Quebec
	• Professional Order of Physiotherapy of Quebec
	• Order of Speech-Language Pathologists and Audiologists of Quebec
	• Canadian Association of Occupational Therapists—Quebec chapter
	• Quebec Physiotherapy Association
	• Quebec Association of Speech-Language Pathologists and Audiologists
	• Be knowledgeable about current professional practice issues in their profession

### Data Collection Procedures

#### Interviews With Rehabilitation Professionals

We carried out semi-structured, in-depth interviews (60–90 min) in person, by phone, or via Zoom/FaceTime from December 2019 to August 2020 (Mak, Thomas, et al., 2023). No follow-up interviews were conducted.

Interviews consisted of open-ended questions (e.g., “Tell me about a typical day at work”) and were based on an interview guide. Separate interview guides were developed for each group (see Supplemental Files 1 and 2) and were based on existing literature on attrition and retention and concepts from CHAT. For example, interview probes about their managers and team members were derived from CHAT concepts (i.e., community). To ensure that the questions were clear and not overly leading, we gathered feedback from a group of graduate students (many of whom are rehabilitation professionals) and pilot-tested the interview guides with two OTs. As interviews progressed, we also modified certain questions based on our experiences of earlier interviews and ongoing data analysis.

All interviews were recorded and transcribed verbatim. Each transcript was verified by the principal investigator (SM) or a research assistant (BM, TO, and NK). More details on the data collection procedures are provided in Mak, Thomas, et al. (2023).

#### Focus Groups With Managers, Professional Associations and Regulatory Bodies, and Professional Education Programs

We conducted focus groups from January to June 2022 by Zoom. Each focus group included 3–6 participants. Focus groups were favored to facilitate reflection and discussion among participants.

Our research team developed the preliminary version of the focus group guides. There were separate focus group guides for each group based on existing literature on attrition and retention, interview findings, and CHAT. Examples of focus group questions and prompts are provided in Supplemental File 3. The questions were aligned with CHAT. For instance, when we asked focus group participants about what positively influences retention of rehabilitation professionals, our probes included questions about relationships with colleagues (community) and institutional processes (rules). We then presented the focus group guides to a group of graduate students (many of whom are rehabilitation professionals), whose feedback was integrated into the final versions.

As focus groups progressed, we modified certain questions. For example, following the first focus group with managers, we realized that two questions elicited similar responses, and therefore, we removed one question for the second focus group of managers.

All focus group sessions were recorded and transcribed verbatim. Each transcript was verified by the first author (SM).

### Ethics

These studies were reviewed and approved by the McGill Faculty of Medicine and Health Sciences Review Board (A02-E12-19A). All interview and focus group participants provided written informed consent to participate in the study (including the audio-recording of the interview or focus group).

### Data Analysis

We began separate data analyses of the interviews and focus group data using NVivo (Version 1.7.1), as soon as the respective recordings were transcribed and reviewed for accuracy. We created a synopsis of each interview and referred to focus group notes to facilitate recall of the interview and focus group content during data analysis, such as how a portion of text related to a participant’s narrative. For French transcripts, SM re-read selected interview and focus group quotes to confirm that they aligned with the codes and consulted with a native Quebec French speaker to ensure accurate interpretation of the participant’s narrative.

We used deductive and inductive approaches and constant comparative techniques (Thorne, 2016). Our inductive approach for the interviews and focus groups included asking questions such as: “What is this about? What is going on here?” while we assigned labels to sections of text. After coding one transcript, AT and MH (qualitative researchers and content experts) reviewed the codes from that transcript and provided feedback. The codes were subsequently modified, and a provisional codebook was created. This step was completed separately for the interview and focus group data. We applied the provisional codebooks to two more transcripts and added several new codes. AT and MH then reviewed the updated codebooks and provided additional feedback. The revised codebooks were subsequently presented to the same group of graduate students who reviewed the interview and focus group guides for feedback and were revised again.

We continued participant recruitment for the interviews until the point where we judged the analytic structure to be stable and that additional interviews were unlikely to lead to significant alterations of this structure. For the focus groups, recruitment ended due to limited stakeholder availability during the COVID-19 pandemic.

Attention to existing literature contributed to our deductive approach. For instance, an important finding from the scoping review on attrition and retention was the unmet needs of PTs in later career stages (Australian Physiotherapy Association, 2013). Awareness of this finding helped us attend to specific codes for the interviews (e.g., *life stage*) and focus groups (e.g., *career longevity*). While both codes were used less frequently (*life stage*—four times, *career longevity*—twice) than other codes, we examined the data to identify how the unmet needs of PTs in late career stages were discussed in the later stages of data analysis, given the importance of this finding.

Drawing from concepts of CHAT also supported the deductive approach. For example, when we reflected on *institutional culture* from the focus group data, we considered how it could shape institutional procedures (rules), the different professional roles (division of labor), and teamwork (community). We also reflected on how a code such as *management structure* might be captured in the visual representation of CHAT; management structure is not an explicit component of CHAT but rather it might be reflected in other components of CHAT such as rules, community, or division of labor. However, to avoid overreliance on the theoretical scaffolding from CHAT and to align with ID, we began with an inductive approach in the earlier stages of data analysis and sought feedback from our wider research team.

We used this analytical structure and drew from CHAT for our combined analysis of the interview and focus group data, where we looked for convergences of codes, while being attentive to divergences, to build categories. We used display tables to organize our codes and categories, from which we developed interpretive themes across and within data sources. For each interpretive theme, we described the scope and the corresponding categories and quotes. We also used concept maps to lay out the themes visually; this process allowed us to identify relationships between themes and to further develop the analytical structure. We then gathered additional feedback from the research team and refined the analytical structure. Using the interpretive themes, we reflected on how these could be operationalized as retention strategies, which helped us to articulate them as sets of retention strategies.

### Reflexivity

The positionalities of our research team members informed our entire research process. Our research team is composed of two OTs, one PT, one S-LP, and one physician. The first author, SM, is an OT with over 15 years of clinical and academic experience in a Canadian OT educational program. Her experiences helped to shape the interview and focus group questions and how she interpreted her findings. AT (OT) is an experienced qualitative researcher in health professions education and knowledge translation. During data analysis, she noted the parallels between her work on the role of context on professional competencies and professional agency, and this study. Her reflections made her aware of how her work influenced the way she viewed the study’s findings. MH is an expert qualitative researcher whose research foci include ethics, health policy, and rehabilitation. His research experiences attuned him to the realities of working with systems and the challenges faced by rehabilitation workers in general which shaped how he viewed the findings. KR is an S-LP who practices in the public health care system and teaches in a Canadian S-LP educational program; her clinical and academic experiences brought a particular attention to the S-LP profession and helped to highlight the commonalities and differences between S-LP and other rehabilitation professions. Finally, SR is a physician, an academic, and researcher in equity and diversity issues in health professions education. His experiences helped us to attend to the structural aspects of health care systems and their impact on health care practices during data analysis.

In addition to reflecting on our positionalities, we used reflexive practices such as memoing throughout the study. For instance, SM noted emotional reactions, thoughts, and potential relationships between ideas that she had during data collection and analysis. In these moments, she reflected on her responses and how they connected to her experiences (e.g., feeling angry about practitioners having to work with few resources). Hence, our reflections, findings from our scoping review, and insights drawn from the existing literature contributed to our research processes (e.g., decisions related to participant recruitment).

## Findings

### Interviews With Rehabilitation Professionals

Fifty-one individuals were interviewed, divided between two groups: (1) attrition (*n* = 14) and (2) retention (*n* = 37). Sixty-three percent were OTs (*n* = 32), 22% (*n* = 11) were PTs, and 16% (*n* = 8) were S-LPs. Participants’ socio-demographic characteristics are summarized in Table 3.

**Table 3.** Interview Participant Characteristics.

	OT	PT	S-LP
Gender			
Male	2	2	1
Female	30	9	7
Other	0	0	0
Geographical location			
Urban	24	7	7
Suburban	8	3	1
Rural	0	1	0
Years of experience			
1–5 years	8	1	5
6–10 years	9	1	0
11–15 years	4	5	2
16+ years	11	4	1
Practice sector			
Public	19	4	2
Private	9	4	1
Other (e.g., schools)	4	2	5
Practice settings			
Hospital-based	15	4	3
Community-based	3	1	0
School settings	0	1	5
Private practice	1	4	0
Other (academic, corporate)	3	1	0
Practice area			
Adults, physical medicine	13	7	2
Adults, mental health	0	0	0
Adults, all	3	0	0
Children, physical medicine	1	1	1
Children, mental health	1	0	1
Children, all	8	1	4
Other (management, corporate, academia, all clientele)	6	2	0

### Focus Groups With Managers, Professional Associations and Regulatory Bodies, and Professional Education Programs

Sixteen people participated in four focus groups: (1) four managers from the private health care sector; (2) three managers from the public health care sector; (3) three representatives from professional associations and regulatory bodies; and (4) six representatives from professional education programs. Table 4 presents the focus group participants’ characteristics.

**Table 4.** Focus Group Participant Characteristics.

Focus Group	Total Number of Participants	Physical Therapy	Occupational Therapy	Speech-Language Pathology
Professional education programs	6	3	2	1
Professional associations and regulatory bodies	3	1	1	1
Private sector managers	4	2	1^a^	1
Public sector managers	3	1^a^	1	1
Total	16	7	5	4

^a^Represents other professionals as well.

### Qualitative Analysis

We identified five sets of retention strategies from the interview and focus group data (Table 5). They represent a spectrum of individual-level (respond to a rehabilitation professional’s needs and desires) to system-level retention strategies (target the environments/systems which a rehabilitation professional may be part of).

**Table 5.** Sets of Retention Strategies With Associated Categories and Codes.

Levels Targeted by Retention Strategies	Sets of Retention Strategies	Categories	Examples of Codes (Reflected in Article)
Individual level	Ensuring that work aligns with values	Practitioner values	Clinical challenges
			Professional identity
		Recognition of practitioner’s work	Career advancement opportunities
			Perceived impact of work
	Improving alignment of work parameters with needs and desires of rehabilitation professionals	Nature of work	Intensity and amount of work
			Diversity of work tasks and responsibilities
		Resources for work	Availability of resources
System level	Modifying physical, social, cultural, and structural aspects of a workplace	Physical	Organization size
		Structural	Management structure
			Barriers to practice
		Cultural	Institutional culture
		Social	Apathy
	Addressing how the profession is governed	Visibility and recognition of profession	Matching salaries to educational degree
			Lack of public awareness
			Expanding roles and responsibilities
		Improving professional practice	Assistants for rehabilitation professions
		Relationship with regulatory bodies	Communication with rehabilitation professionals
Both	Offering informal and formal benefits	Formal benefits	Social benefits
			Professional development funds
		Informal benefits	Mentorship
			Schedule flexibility
			Professional growth opportunities

Below, we describe two sets of individual-level strategies, *ensuring that work aligns with values* and *improving alignment of work parameters with needs and interests of rehabilitation professionals*, and two system-level strategies, *modifying physical, social, cultural, and structural aspects of a workplace* and *targeting how the profession is governed*. We then discuss a final set of strategies, *offering informal and formal benefits*, that are at the intersection of individual- and systemic-level retention strategies.

Verbatim excerpts in French have been translated into English.

### Individual-Level Retention Strategies

#### Ensuring That Work Aligns With Values

This strategy underscores the importance of ensuring that the attributes of a rehabilitation professional’s work match their values. For example, an S-LP with over 15 years of hospital-based experience described how she values professional growth. Thus, having occasional challenges in her work stimulates her to acquire new knowledge and to fulfill this value: “every now and then you have a case that just surprises you and that makes you go and research things, and … allows you to … continue to grow” (retention, S-LP6)*.*

Participants in the professional associations and regulatory bodies focus group added how they value growth in the profession. They described that clinical challenges help clinicians to innovate, explore new clienteles and practice areas, seek new knowledge, and, ultimately, use their skills to their fullest. Therefore, these clinician-driven initiatives contribute to advancing their professions.

Having an impact on one’s team and on clients was valued by half of interview participants and discussed in all focus groups. However, participants in the professional education programs focus group reported that a rehabilitation professional’s perceived impact of their work is validated when their clients and their employer recognize their work (U2). For instance, an OT with 7 years of experience in private practice identified career advancement opportunities as one way to provide this recognition:

We hear stories of people who have been in companies for a long time and then ask for advancements, ask to be recognized for certain extra tasks they do and then they never have this recognition. Well, it should come as no surprise that at some point these professionals leave because this is basic. (Retention, OT3)

Participants also valued regular interaction with their profession-specific community, to reinforce their professional identity. This was raised by eight interview participants (mostly OTs) and by the professional education program focus group. A participant with over 30 years of experience described:

I had a bit of an identity crisis […] I felt like I was living in the shadow of the physios. […] Once I was in rehab and I was working in a huge OT department with a lot of really strong OTs that knew who they were, ahh, I felt so strong as a member of the interdisciplinary team more so than I had felt at the [NAME]. (Retention, OT4)

Overall, participants discussed the importance of having opportunities to grow professionally, to connect with others from their profession, and to receive validation for the impact of their work. Identifying what a rehabilitation professional values and ensuring alignment between those values and what the work offers may foster more positive work experiences.

#### Improving Alignment of Work Parameters With Needs and Interests of Rehabilitation Professionals

This set of individual-level retention strategies focused on practical and structural features of the work and how they align with the needs and interests of rehabilitation professionals. Elements included (1) the intensity (e.g., urgency of one’s role) and amount of work (number of clients); (2) diversity of work tasks and responsibilities; and (3) availability of resources. These aspects were deemed essential by interview participants for managing their well-being and their personal and work responsibilities. Though half of the focus groups also discussed these particular work characteristics, there was greater emphasis on the diversity of work tasks; a participant from the private sector managers focus group highlighted how this diversity of work offers opportunities for learning: “I think a big part of it is variety, opportunity to learn, opportunity to control and develop into specific types of caseloads” (managers—private sector, PRIM1).

The importance of such alignment is highlighted by this PT (licensed for 15 years, private sector). She identified a selected number of patients per hour to characterize a high workload (e.g., 15 patients): “Yes, the ratio [of three patients in an hour], and whether you want it or not, the higher the ratio, the more your quality of treatment decreases. So, that’s what I find very demanding about our job” (retention, PT2). The participant used a ratio to illustrate that the expected number of patients seen per hour was unmanageable for her and compromised the quality of care when she worked in the public sector.

Thus, the focus of this individual-level retention strategy is to ensure that work characteristics align with a rehabilitation professional’s capacity and that the work characteristics (e.g., workload) remain manageable so that they do not negatively impact quality of patient care.

### System-Level Retention Strategies

#### Modifying Physical, Social, Cultural, and Structural Aspects of a Workplace

Focus group participants (managers from private and public health sectors) discussed how retention strategies should target the attributes of a workplace. Private sector managers discussed organization size as an important consideration insofar as the nature and type of supports offered to their staff are concerned: “I think [it] makes a huge difference because we can take that time, you know. And we’re not so huge yet” (managers—private sector, PRIM3).

In contrast, managers in the public sector identified the management structure and institutional culture as critical for fostering retention. One participant described how as a rehabilitation professional herself, she understands other professionals’ daily realities but struggles to support other groups of professionals whose realities are different from her own (e.g., social workers):

I think one of the key points would be having a structure of management of people who understand them, can help them, and support them in the right way. […] I’m doing a pretty good job with my OTs and my PTs, but everybody else, I know that I just can’t support them the right way. (Managers—public sector, PUM2)

Nearly all interview participants reported that a supportive work environment was key to retention. In particular, participants emphasized access to mentorship and communities of practice in order to develop new knowledge and skills in an unfamiliar clinical environment (e.g., new graduates) and to maintain one’s abilities to practice in an existing one. A PT with 15 years of experience gave the following example:

We just hired a new grad […] he just needs a lot of nurturing at the beginning so he feels comfortable in this environment […] because the last placements he was working at, he was in a pediatric setting and a geriatric setting […] I want him to feel comfortable basically and not worry about his expertise. That, we will just work on those and grow those. (Retention, PT7)

Several interview participants also shared their perception of a lack of agency among their peers (social aspect of workplace) and their concern for the future of their profession: “I have the impression that there is a lack of initiative in OT to protect the profession, to develop it” (retention, OT5).

A third of interview participants described a need for managers to reduce rather than contribute to structural barriers to their practice (e.g., delays in obtaining equipment). A PT with over 15 years of experience in private practice explained how a manager’s decision to change their clinic’s location added challenges to collaborating with referring physicians who had previously been nearby:

He [manager] has moved us from one location to another within the same building and without really understanding how that can either impact our practice […] it can decrease our interactions with the doctors, which is important. (Retention, PT1)

Interview and focus group participants emphasized how the elements of a workplace can shape a rehabilitation professional’s experience of work, both positively and negatively. In turn, the nature of such experiences can influence their desire to stay in or leave their profession.

#### Addressing How the Profession Is Governed

This set of system-level retention strategies targets the organizations and laws that govern the practice of the profession. This was mainly discussed among professional associations and regulatory body participants. They underscored how salaries of rehabilitation professionals need to be better calibrated with the level of education required for entry to practice. For example, one participant expressed that some PTs are paid as if they have an undergraduate professional degree rather than a master’s degree (professional associations and regulatory bodies, POA1). Expanding roles and responsibilities of rehabilitation professionals was also identified as an important strategy to optimize a profession’s field of expertise and to foster professional retention.

It is to seek out our entire field of expertise as much as possible. It may be pursuing advanced practice. […] Maybe it will encourage people to stay in their profession. (Professional associations and regulatory bodies, POA1)

Participants in this focus group also drew attention to the need for professional regulatory bodies to provide direct, clear, and concise communication to their members, and to offer opportunities for their members to connect with one another (professional associations and regulatory bodies, POA2).

Contrary to professional association and regulatory body participants, interview participants identified different priorities related to governance. Approximately half of the interview participants viewed the public’s lack of awareness of their profession as a key priority; increasing public awareness of their added value could, in their view, improve retention. Participants also voiced a need for greater involvement from professional education programs in partnership with professional associations and regulatory bodies, to address this lack of awareness. For example, an OT working in pediatrics for 20 years spoke about the need to advocate to the government for greater involvement of OTs in schools for early child development (retention, OT8).

The creation of assistant positions for the rehabilitation professions (e.g., OT assistant) was raised by participants during interviews and one focus group (managers—public sector). A few participants discussed how daily clinical work can be repetitive and filled with mundane tasks that do not require a high level of expertise. A participant expressed that:

[…], from a very pragmatic level, and very down to earth would be getting OT assistants, so that we can separate … [tasks] that we were trained to do the things that could be done by others. (long pause) So … to me that would target more the skills of the OTs and probably challenge them more and … increase motivation, in the workplace. (Retention, OT25)

This view was also shared by public sector managers (managers—public sector, PUM2). The inclusion of assistants was viewed as a viable strategy to support retention and to lessen the impact of the workforce shortages in the rehabilitation professions.

### Strategies That Encompass Individual-Level and System-Level Approaches

#### Offering Informal and Formal Benefits

Most interview and focus group participants identified several formal and informal benefits offered by health care institutions that support professional retention. Formal benefits are offered publicly by employers, such as social benefits and funding for professional development. In contrast, informal benefits are negotiated privately between a rehabilitation professional and their manager. Formal and informal benefits simultaneously target two levels of a system: (1) individual, such as a rehabilitation professional’s needs (individual level); and (2) broader, systemic levels, such as a health care institution or network (system level).

Participants provided examples of informal benefits including access to mentorship, flexibility in one’s schedule, and other professional growth opportunities (e.g., involvement in special projects). A PT with 15 years of experience explained how a trusting relationship with her manager creates an implicit understanding that she will make up time if she has to leave early (informal benefit):

But also in terms of flexibility, say I need to go to the dentist at 4 o’clock, I can leave work at 3:30 and I don’t even have to tell my boss. I just do it and make up the hours another day. And that’s because they trust me. (Retention, PT3)

The professional-manager relationship was also discussed in the private sector managers’ focus group. A PT clinic owner talked about how knowing her employees’ goals helped her to support their career growth: “It’s being aware of each person’s career development plan. It goes hand-in-hand with the close relationship between managers and professionals. […] We can better accompany them by having a closer relationship and following through with this person’s plan” (managers—private sector, PRIM4).

In summary, participants described how informal and formal benefits contributed to retention. A close and trusting professional-manager relationship was critical to accessing informal benefits.

## Discussion

This paper presented the perspectives of 51 rehabilitation professionals and 16 representatives from four stakeholder groups, namely, managers, professional education programs, professional associations, and regulatory bodies, about potential retention strategies. We used CHAT to reflect on health care and educational systems and their relationships with other systems (e.g., professional regulation). Thus, our use of CHAT helped to identify different sets of retention strategies and their targeted level of intervention.

There were five sets of retention strategies targeting rehabilitation professionals’ needs and desires, and the environments and systems that rehabilitation professionals engage with. However, when reflecting on CHAT, it became evident that all strategies involve to varying degrees rehabilitation professionals’ managers and institutions (community), and more broadly, the health care, educational, and professional systems (activity system). The involvement of these parties reflects the levels of intervention described in the retention literature (individual/leader, group, and organizational) (Fox et al., 2022). Below, we draw on the literature pertaining to individual-level retention strategies to explore their relevance to our study findings.

Individual-level retention strategies are aimed at making changes at the level of the professional or leader, such as a manager learning new ways to organize their team (Fox et al., 2022). In our study, many interview and focus group participants underscored how a close and trusting professional-manager relationship supports retention (Mak, Thomas, et al., 2023). The importance of the professional-manager relationship is further supported in the literature (Silver et al.). In a paper about retention strategies for rehabilitation professionals, physicians, and nurses, Silver et al. (2024) emphasize the need for a supportive organizational culture, authentic and ethical leadership, and genuine appreciation of professionals from supervisors. All these aspects of the professional-manager relationship could be conceived as pull factors toward other health environments. An overemphasis on individual/leader-focused interventions may, however, be problematic in cases where a manager has limited decision-making power due to broader organizational or systemic factors (Elliott et al., 2016). This dynamic is especially evident in the public health sector where managers may be constrained in their ability to nurture such relationships given prevailing institutional cultures that prioritize institutional needs over the needs of health workers. Regulatory bodies’ decisions about assistants can broaden or restrict the pool of human resources available for managers to hire. Therefore, these examples demonstrate a need for retention strategies targeting the systemic barriers that substantially undermine managers’ and rehabilitation professionals’ desire for change in the health care system (Kroezen et al., 2015).

A limited sense of professional agency was also evident in the interviews with rehabilitation professionals, many of whom felt far removed from decisions about retention strategies. The impact of systemic factors on professionals’ sense of agency has been discussed in the literature (Thomas et al., 2023). A recent scoping review by Thomas et al. (2023) on the influence of contextual characteristics on professional competencies identified six factors including leadership and agency. Agency contained six dimensions which potentially influence a health professional’s practice: (1) opportunity for autonomy about work/clinical decisions; (2) opportunity for autonomy about scope of practice; (3) opportunity for empowerment; (4) control of work duties; (5) control of time; and (6) participation in decision-making (Thomas et al., 2023). Though dimensions of agency resonate with our participants’ desires to seek alignment between their priorities and their work characteristics, there are few opportunities to participate in decision-making regarding their work needs. One may ask why this is so and how this may change. Denis et al. (2021) argued that multiple health system reforms and budget cutbacks have hindered the integration of participatory and inclusive decision-making practices in Quebec’s health care system. The authors also added that it is time for these practices to be implemented to adequately address workforce shortages (Denis et al., 2021). Thus, a first step can be at an institutional level, where institutions create opportunities whereby managers and administrators seek feedback from rehabilitation professionals on resources and programs for their work (Saks, 2022).

Opportunities for dialogue between clinicians and managers have been suggested to foster work engagement among nurses and help them feel that their perspectives matter (Holland et al., 2017). However, a plausible obstacle to such opportunities is the limited participation of rehabilitation professionals, in part due to their limited agency (Walton, 2020). Therefore, before involving rehabilitation professionals at broader levels of decision-making, managers and administrators need to identify the conditions (e.g., liberation of clinical duties) to enable rehabilitation professionals to rediscover their sense of agency. In turn, having optimal conditions for engagement in activities outside of clinical work may be key to fostering active participation from rehabilitation professionals.

Work redesign is an example of an organizational intervention which aims to change systemic practices and policies by influencing the work environment. This approach can challenge structural aspects of workplaces by reorganizing work tasks and changing work conditions and environments to support rehabilitation professionals’ well-being (Fox et al., 2022). Organizational interventions have been shown to be more effective than individual or group ones (Teoh et al., 2023). In our study, organizational interventions were reflected in this set of system-level strategies, *modifying structural aspects of workplaces*, and aligned with the structure of CHAT. From this perspective, adapting an activity system to influence its components (i.e., subjects) is likely more effective to influence the activity system rather than changing one of its components. Implementing flex work, self-scheduling, and lean management practices (optimizing value for patients) would be concrete ways for institutional or systemic changes, instead of making changes at the level of the professional (Fox et al., 2022). However, changing an institutional culture is a difficult undertaking and could be facilitated with governmental and/or institutional buy-in over an extended period of time (Kroezen et al., 2015). Given the positive outcomes derived from organizational interventions (e.g., increased job satisfaction and improvement in attrition) (Linzer et al., 2015), they may be a worthwhile avenue to explore to address professional attrition in the rehabilitation professions.

Interview participants emphasized the need to address public awareness of their profession, through partnerships across different systems. However, intersectoral partnerships that extend across health care (clinical sites), education (professional education programs), and the professional system (professional associations and regulatory bodies) can be challenging to operationalize, requiring authentic collaborative decision-making processes, shared goals, and mutual understanding from all (Anaf et al., 2014; Lown et al., 2019). Using CHAT in this instance can be key to identifying commonalities between systems as a way to move toward a concerted effort: a close examination of how a system’s rules impact positively on their partnerships could be a possible starting point. CHAT can also highlight how the systems themselves pose challenges to intersectoral collaboration; organizational culture and rigidities, contested planning priorities, and professional attitudes are some of the many barriers to the successful implementation of intersectoral partnerships (Anaf et al., 2014). However, the actors of these systems need to learn about each other’s realities and be willing to compromise their own priorities if they hope to improve current health workforce shortages (Kim et al., 2024); otherwise, these actors will continue to work in silos and perpetuate retention issues in the rehabilitation workforce.

There were notable differences in the retention strategies reported by focus group participants, in terms of their priorities, and the level of interventions (individual vs. systemic). Participants from the professional education programs described systemic interventions to improve fieldwork opportunities to optimize graduates’ preparedness for entry to practice (educational system), while managers directed their efforts to individualized supports and resources for clinical practice. Reflecting on these differences using the structure of CHAT draws attention to how professional education programs and managers are situated differently within their own system. A professional education program can be viewed as an activity system, as compared to managers who are situated within the community of a different system (i.e., health care). Therefore, it makes sense that being a component within a system versus a system itself entails different levels of intervention. An intervention aimed at changing a component of a system will need to consider how other components of that system may be affected by that intervention and vice versa. On the other hand, an intervention that targets a system will likely bring change to all components of that system, creating a greater impact.

Drawing from CHAT, we noted that participants from the professional associations and regulatory bodies focus group identified systemic interventions aimed at multiple systems (health care and professional systems). Examples of such interventions were aligning salaries to the educational degree obtained by rehabilitation professionals and expanding roles of rehabilitation professionals; both of which were identified as contributors to retention in our scoping review (Mak, Thomas, et al., 2023). This finding underscores the substantial impact that multi-systemic interventions can have on professional retention, in contrast to interventions focused on a single system or an individual (Teoh et al., 2023).

Participants from the private sector focus group appeared confident about their capacity to address their employees’ needs. In fact, these participants described ways they supported their employees (e.g., one-on-one involvement with rehabilitation professionals). These findings point to differences in management structures in private and public health sectors, where managers’ decision-making power in the public sector is more limited by systemic influences, a point raised earlier in the discussion. It may also reflect these participants’ limited time to interact with their employees in the public sector. However, we also acknowledge that private sector managers are influenced by their clinic’s profitability (Hudon et al., 2019) and thus are driven to support employee retention so that clients can be seen. Nonetheless, these findings are important contextual differences as they have implications for the design of retention strategies (Mak, Thomas, et al., 2023), but also for the possible movement of rehabilitation professionals from the public system to the private sector. This migration across health sectors has already been observed in physicians due to financial incentives and better working conditions (El Koussa et al., 2016). To improve the workforce capacity of the public health care system, better incentives will be needed to foster the professional retention of rehabilitation professionals.

### Strengths and Limitations

This study had three main strengths. Using CHAT as the theoretical basis of our study helped to guide study decisions and data analysis for the interviews and focus groups. However, to remain consistent with ID and provide novel insights on the topic of attrition and retention, we also developed our findings beyond the structure and concepts of CHAT. A second strength was that the interview and focus group participants were recruited from various sectors (private and public health sectors, professional associations and regulatory bodies, and educational sector), which diversified the participants in our study. Finally, we also recruited participants from all three professions which ensured that profession-specific perspectives were present.

We had aimed for 4–6 participants for each focus group, but two of our focus groups (professional associations and regulatory bodies, and public sector managers) had fewer than our targeted group size (three participants). Focus group participants were difficult to recruit due to the high workload associated with the COVID-19 pandemic. Therefore, the smaller group size may have led to less productive discussions and did not allow us to achieve purposeful sampling. For the interviews, we also had difficulty recruiting rehabilitation professionals who left their profession (*n* = 14) compared to the retention group (*n* = 37), as they were difficult to locate. For both interviews and focus groups, we used multiple strategies to advertise our study, including email, social media platforms, and word of mouth (snowball sampling); however, despite our efforts, participant enrolment was limited. The lower number of participants in the attrition group (interviews) and the focus groups may limit our ability to make comparisons and conclusions due to the imbalances within the sampling.

## Conclusion

Attrition and retention are key factors for a health care system’s capacity to offer services to a population. We have presented key retention strategies, which range from individual-focused to organizational/systemic interventions, obtained from interviews with 51 rehabilitation professionals and focus groups with 16 members of four stakeholders. We have also highlighted how multi-systemic interventions may exert a stronger influence than those directed at one system or an individual, and the pressing need for an intersectoral approach to addressing retention. We hope that these findings will help to orient stakeholders in developing targeted retention strategies for rehabilitation professionals. Directing efforts toward interventions to reduce attrition and support retention will be key to ensuring that health care systems have the workforce capacity to respond to current and future rehabilitation needs.

## Supplemental Material

Supplemental Material - Stakeholder Perspectives on Retention Strategies for Rehabilitation Professionals: A Qualitative Study Supplemental Material for Stakeholder Perspectives on Retention Strategies for Rehabilitation Professionals: A Qualitative Study by Susanne Mak, Matthew Hunt, Saleem Razack, Kelly Root, and Aliki Thomas in Qualitative Health Research
