# Supplementary material for: Stakeholder Perspectives on Retention Strategies for Rehabilitation Professionals: A Qualitative Study
Source: Qual Health Res. 2024 Dec 12;35(14):1579–93. doi: 10.1177/10497323241286387 (PMC12552756; doi:10.1177/10497323241286387)
Supplement: Supplemental Material - Stakeholder Perspectives on Retention Strategies for Rehabilitation Professionals: A Qualitative Study [file sj-pdf-1-qhr-10.1177_10497323241286387.pdf]

Supplemental file 1: Retention interview guide questions and prompts

| **Interview question** | **Probes** |
|---|---|
| 1. To start, please tell me about a typical day at work. | • What is your role as an OT, PT or S-LP?<br>• Who are the other members of your team? What are their roles?<br>• What is your role on this team?<br>• Describe other individuals that impact your role or work, but who are not part of your team. |
| 2. Briefly describe what it means for you to be an OT, PT, or S-LP. | • What motivates you to go to work every day?<br>• What do you find most satisfying about being an OT/PT/SLP?<br>• What makes you not want to go to work?<br>• What other tools, resources, etc..make your job easier?<br>• What tools, etc…do not? |
| 3. What other environments or factors influence your practice? | • Home, university environment?<br>• Other responsibilities or roles outside of your work? |
| 4. Is there a university associated with your clinical site? If so, what are the university's expectations of the clinical site? And vice versa? | • How does the expectations from that relationship affect you and your daily work? |

| | |
|---|---|
| 5. What do you love about your work? | • What aspects of your work makes you want to stay in the profession?<br>• Are there other factors outside of your job that affects your decision? |
| 6. What don't you like about your work? | • How does it (the things you don't like about your work) impact affect your decision to stay/leave?<br>• Did you ever consider leaving your profession? If so, what made you change your mind? |
| 7. How could health care environments help retain OTs/PTs/S-LPs? | • What strategies could be used? Role of the OT/PT/S-LP? Support or resources for the job? Work schedule?<br>• Who would be involved? Other members of your team? Managers? Other members of the profession?<br>• What do you foresee as potential obstacles to these retention strategies? |
| 8. Do you think university programs (in OT/PT/SLP) play a role in retaining rehabilitation professionals in their professions? If so, how? | • What could be potential challenges to these strategies? |

| | |
|---|---|
| 9. Where do you think professional regulatory bodies/associations fit into the retention of rehabilitation professionals? | • Resources to support your work, like mentoring, discussion forums, continuing education courses?<br>• Advocating for changes in reserved acts (e.g. no MD prescription needed for mobility aids – prescribed by OT/PT)?<br>• Promoting OT/PT/S-LP in the general public? |

Supplemental file 2: Attrition interview guide questions and prompts

| **Research question** | **Probes** |
|---|---|
| 1. To start, please tell me about your current employment. | • Where do you work?<br>• In which field?<br>• What does a typical day look like? |
| 2. Tell me briefly about your past experiences working as an OT, PT or S-LP. | • Where did you work?<br>• What type of clientele did you work with?<br>• How long did you work in this/these position(s)?<br>• What made it hard for you to go to work? |
| 3. What did you love about your work? | • What aspects of your work made you want to stay in the profession?<br>• Were there other factors outside of your job that affected your decision? |
| 4. Tell me about how you came to your decision to leave your profession.<br>Stopped being a member | • How were these work experiences related to your decision?<br>- Institutional processes, rules or policies influence your decision? (health care environment)<br>- Tools, resources, etc…(health care environment)<br>- The actual daily work (responsibilities, tasks)? |

| | |
|---|---|
| | • How did your employer respond when you shared your desire to leave your profession?<br>• What about your colleagues? |
| 5. How did health care environments influence your decision to leave the profession? | |
| 6. Did university programs in OT/PT/S-LP play a role in you leaving the profession? If so, how? | |
| 7. Other than university programs and health care environments, how did professional regulatory bodies/associations influence your decision to leave? | • Expectations from professional regulatory bodies? E.g. charting, continuing education courses, etc..<br>• Support for professional practice? |
| 8. What about other experiences (e.g. home) outside your work? How does those factor into your decision to leave your profession? | • How did other interests, commitments or responsibilities contribute to your decision-making? Family/personal life, etc.. |
| 9. What could have changed your mind? | • Consider the three groups/environments we spoke about:<br>a. Health care environments<br>b. University programs in OT/PT/S-LP |

| | |
|---|---|
| | c. Professional regulatory bodies/associations |

Supplemental file 3: Example of focus group questions and prompts (managers stakeholder group)

| **Question** | **Prompt** |
|---|---|
| 1. Which rehabilitation professionals do you hire in your facilities? | • OTs, PTs, S-LPs?<br>• How many do you have from each profession under your supervision? |
| 2. How often do you hire a new OT, PT or S-LP in your facilities? | • What are the reasons for the new hires??<br>• Have you experienced turnover/change in staff as a problem? |
| 3. What do you think positively influences the reasons why OTs, PTs, S-LPs stay? | • Relationships with other colleagues/team members)?<br>• Institutional processes?<br>• The impact of their role on their patients/clients?<br>• Feeling valued and being recognized for their work? |
| 4. On the flip side of that question, what affects those reasons in a negative way? | |
| 5. Which reasons are specific to each profession? | |
| 6. How do you make sure that your OTs, | |

| | |
|---|---|
| PTs, S-LPs stay? OR How can institutions positively affect retention of rehabilitation professionals? | |
| 7. Do you feel that your employees are satisfied with their work? | |
| 8. Outside of the workplace, what else could affect why your employees have stayed or left? | Other roles that your employees (e.g. parent, student) have which may have affected their decision to stay/leave? |
| 9. What are possible strategies for retention of rehabilitation professionals? | What are possible barriers and facilitators to those strategies? |
| 10. Are there any other comments you would like to share today? | |
